# Multiple heterochronic gastrointestinal stromal tumors in the stomach detected 6 years after resection: a case report

**DOI:** 10.1186/s40792-020-00812-1

**Published:** 2020-03-07

**Authors:** Tadahito Yasuda, Kojiro Eto, Naoya Yoshida, Shiro Iwagami, Yukiharu Hiyoshi, Youhei Nagai, Masaaki Iwatsuki, Takatsugu Ishimoto, Yoshifumi Baba, Yuji Miyamoto, Takuya Shiota, Yoshiki Mikami, Hideo Baba

**Affiliations:** 1 Department of Gastroenterology, Graduate School of Medical Science, Kumamoto University, 1-1-1 Honjo, Kumamoto, 860-8556 Japan; 2 Department of Diagnostic Pathology, Kumamoto University Hospital, Kumamoto, Japan, 1-1-1 Honjo, Kumamoto, 860-8556 Japan

**Keywords:** Multiple heterochronic GIST, Adjuvant therapy

## Abstract

**Background:**

To date, only a few cases of multiple GISTs with different clones in different organs have been published. However, a case of multiple GISTs with different clones occurring in a single organ has never been reported.

**Case presentation:**

A 41-year-old patient underwent laparoscopic partial gastrectomy for gastric gastrointestinal stromal tumor (GIST) in 2012. The pathological findings showed high-risk characteristics for recurrence, so he received adjuvant therapy with imatinib for 3 years. In 2018, 3 years after completing the adjuvant therapy, tumor lesions at residual gastric cardia were incidentally identified by follow-up computed tomography (CT). The pathological findings of the tumor biopsy revealed gastric GIST. He underwent secondary laparoscopic partial gastrectomy and was diagnosed with high-risk GIST. Adjuvant therapy with imatinib was restarted immediately. The two gastric GISTs had the same exon 11 mutations in the c-kit gene, but they had different missense mutations. This molecular heterogeneity suggested that they were derived from different origins.

**Conclusion:**

We reported a multiple heterochronic GIST in the stomach detected 6 years after resection. There may be a possibility that another heterochronic GIST will occur in the remnant stomach in the future, so close follow-up will be needed.

## Background

Gastrointestinal stromal tumors (GISTs) are the most common mesenchymal tumor of the gastrointestinal tract originating from cells of Cajal [1], and the estimated annual incidence is 10 to 20 per million. The standard treatment for a primary resectable GIST without distant metastasis is surgery. Adjuvant therapy with imatinib for 3 years is recommended for high-risk GISTs [2].

GISTs are commonly generated as a solitary tumor [3], and multiple GISTs are relatively rare [4], except in heredity disorders such as von Recklinghausen disease [5] [6] and Carney’s syndrome [7]. In addition, multiple GISTs previously reported synchronously occurred in different organs [8].

Herein, we report a case of multiple heterochronic GISTs with different clones metachronously generated in the stomach. To the best of our knowledge, this is the first case of multiple GISTs with different clones in a single organ.

## Case presentation

**Fig. 1:**
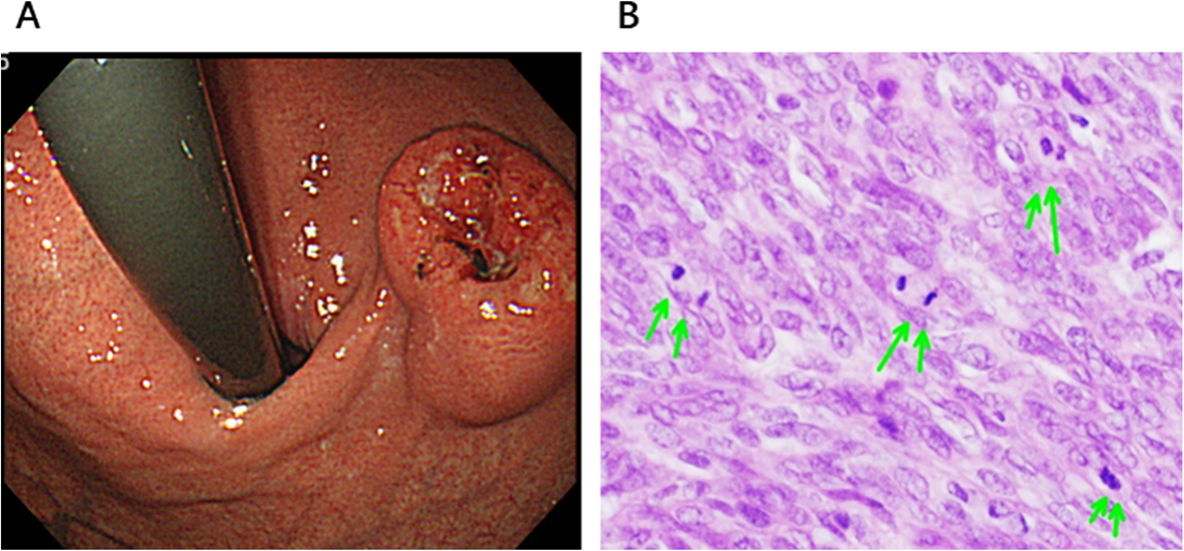
**a** Gastrointestinal endoscopic examination showed a submucosal tumor 35 mm in diameter at the anterior wall of the gastric cardia. **b** Histological examination of the resected specimens showed a nuclear fission image with 50 high-power fields

**Fig. 2:**
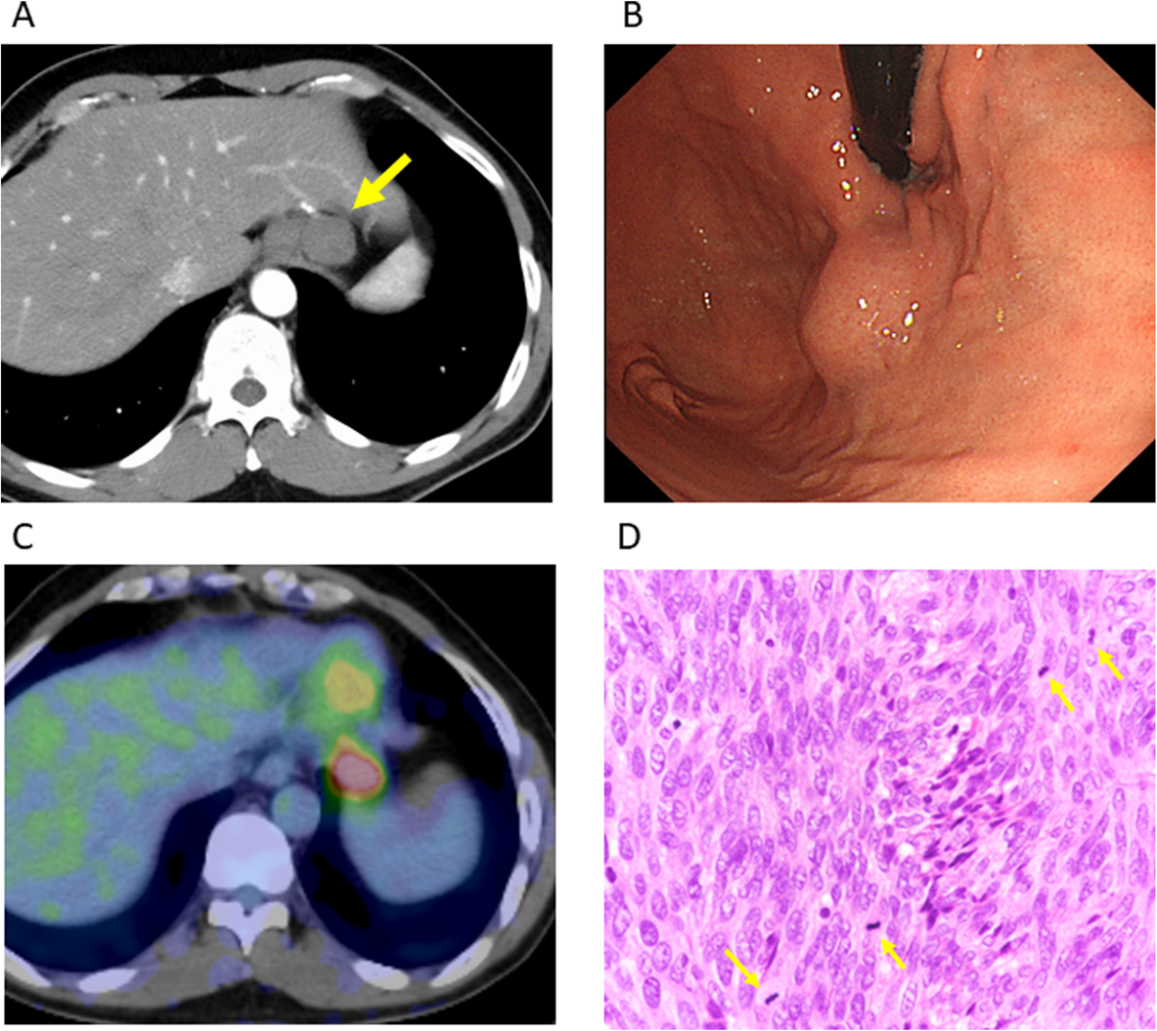
**a** Computed tomography showed a gastric tumor measuring 30 mm in diameter (arrow). **b** Gastrointestinal endoscopic examination showed a submucosal tumor at the anterior wall of the gastric cardia. **c** Positron emission tomography-CT (PET-CT) showed abnormal uptake only at the gastric cardia. **d** Histological examination of the resected specimens showed a nuclear fission image with × 50 high-power fields

A 41-year-old man underwent laparoscopic partial gastrectomy for gastric GIST in 2012. He has no familial history of GIST-rerated heredity disorder. The pathological examination showed the negative resection margins, and there was no heterogeneity. The number of nuclear fissions was 50–70 with 50 high power fields. Therefore, the pathological findings suggested that the tumor had high-risk characteristics of recurrence according to the modified Fletcher classification (Fig. 1a, b). Thus, he received adjuvant therapy with imatinib for 3 years and follow-up computed tomography (CT) every 6 months. No recurrence and new one occurred afterwards. In 2018, 3 years after the completion of adjuvant therapy, tumor lesions were detected at the gastric cardia with a diameter of 30 mm via follow-up CT (Fig. 2a). Laboratory data, including tumor markers, were almost within the normal range. Gastroscopic examination showed a submucosal tumor at the gastric cardia with a diameter of 20 mm (Fig. 2b). The tumor was located away from the resection margin of the previous surgery, which suggested that the tumor was not a local recurrence but a newly generated tumor. Immunohistochemistry of the biopsy from the tumor showed a positive KIT protein, and the tumor was diagnosed as a gastric GIST. Positron emission tomography (PET) CT showed abnormal high uptake at gastric cardia (Fig. 2c).

He finally underwent laparoscopic partial gastrectomy again. The pathological examination showed KIT (+), CD34 (+), desmin (−), and S-100 (−) in the tumor. The number of nuclear fissions was 50–70 with 50 high-power fields. Therefore, the tumor was diagnosed as a high-risk gastric GIST (Fig. 2d).

We conducted a mutation analysis on both gastric GISTs. They had the same exon 11 mutations in the c-kit gene but different missense mutations. The first GIST had the W557_V559 > ST codon mutation of exon 11, whereas the second GIST had the W557_K558 > ST codon mutation of exon 11. This molecular heterogeneity suggested that they were derived from different origins. Therefore, we diagnosed multiple heterochronic GISTs in the stomach. Consequently, adjuvant therapy with imatinib was restarted for the second time.

## Discussion

To date, only a few cases of multiple GISTs with different clones in different organs have been published [8–10]. However, a case of multiple GISTs with different clones occurring in a single organ has never been reported. In the present case, mutation analysis of the c-kit gene showed that the two metachronous GISTs had different missense mutations of exon 11.

In the present case, we diagnosed the second GIST as a second primary GIST with different mutations, not local recurrence. Therefore, we performed surgical resection and administered subsequent adjuvant chemotherapy with imatinib. If a local recurrence was diagnosed, a second agent could possibly be administered. Our case suggested that mutation analysis of the c-kit gene is integral to precisely diagnosing the origin of multiple metachronous GISTs and assembling an optimal treatment strategy, which can affect the long-term outcome [11].

Unidentified genetic and environmental backgrounds may affect the occurrence of multiple GISTs with different clones. Several reports of multiple synchronous GISTs with different clones may imply the existence of risk factors for GIST occurrence in individual cases. Some cases contingently observed multiple microscopic GISTs with different clones in the stomach resected for other diseases and in a cadaver. Agaimy et al. suggested that these cases may involve multiple etiological factors inducing somatic c-kit or PDGFRA mutations to initiate the microscopic GISTs [8, 12, 13]. Based on the information, there is a possibility that multiple GISTs can be predicted in the future.

In the present case, adjuvant imatinib was administered after resection of the first GIST, which might suppress the incidence of a second GIST. This case may also have these risk factors, such as somatic c-kit or PDGFRA mutations for GIST occurrence. Thus, we must pay careful attention to the third primary GIST in the future, notably after the completion of the second round of adjuvant therapy with imatinib.

## Conclusion

We reported a multiple heterochronic GIST in the stomach detected 6 years after resection. Mutation analysis of the c-kit gene is integral to precisely diagnosing the origin of multiple GISTs and assembling an optimal treatment strategy. Moreover, there may be a possibility that another heterochronic GIST will occur in the remnant stomach in the future; thus, close follow-up will be needed.

## Data Availability

Data sharing is not applicable to this article as no datasets were generated or analyzed during the current study.

## Abbreviations

CT: Computed tomography
GIST: Gastrointestinal stromal tumor
PDGFRA: Platelet-derived growth factor receptor alpha
PET: Positron emission tomography

## References

[1] Liegl-Atzwanger B, Fletcher JA, Fletcher CD (2010). Gastrointestinal stromal tumors. Virchows Arch.

[2] Iwatsuki M (2019). Neoadjuvant and adjuvant therapy for gastrointestinal stromal tumors. Ann Gastroenterol Surg.

[3] Nilsson B (2005). Gastrointestinal stromal tumors: the incidence, prevalence, clinical course, and prognostication in the preimatinib mesylate era--a population-based study in western Sweden. Cancer.

[4] Li K (2019). Multiple gastrointestinal stromal tumors: analysis of clinicopathologic characteristics and prognosis of 20 patients. Cancer Manag Res.

[5] Takazawa Y (2005). Gastrointestinal stromal tumors of neurofibromatosis type I (von Recklinghausen’s disease). Am J Surg Pathol.

[6] Sawalhi S (2013). Behavior of advanced gastrointestinal stromal tumor in a patient with von-Recklinghausen disease: case report. World J Clin Oncol.

[7] Carney JA (2009). Carney triad: a syndrome featuring paraganglionic, adrenocortical, and possibly other endocrine tumors. J Clin Endocrinol Metab.

[8] Tokunaga M (2018). Multiple synchronous sporadic gastrointestinal stromal tumors in the stomach and jejunum. Intern Med.

[9] Comandini D, Damiani A, Pastorino A (2017). Synchronous GISTs associated with multiple sporadic tumors: a case report. Drugs Context.

[10] Gasparotto D (2008). Multiple primary sporadic gastrointestinal stromal tumors in the adult: an underestimated entity. Clin Cancer Res.

[11] Agaimy A (2009). Multiple sporadic gastrointestinal stromal tumours arising at different gastrointestinal sites: pattern of involvement of the muscularis propria as a clue to independent primary GISTs. Virchows Arch.

[12] Kawanowa K (2006). High incidence of microscopic gastrointestinal stromal tumors in the stomach. Hum Pathol.

[13] Agaimy A (2007). Minute gastric sclerosing stromal tumors (GIST tumorlets) are common in adults and frequently show c-KIT mutations. Am J Surg Pathol.

